# Similar function and complications for patients with short versus long hip nailing for unstable pertrochanteric fractures

**DOI:** 10.1051/sicotj/2018023

**Published:** 2018-06-15

**Authors:** Ioannis P. Galanopoulos, Andreas F. Mavrogenis, Panayiotis D. Megaloikonomos, Christos T. Vottis, Evanthia Mitsiokapa, Panayiotis Koulouvaris, Dimitrios S. Mastrokalos, Panayiotis J. Papagelopoulos, Vasilios A. Kontogeorgakos

**Affiliations:** First Department of Orthopaedics, National and Kapodistrian University of Athens, School of Medicine, ATTIKON University Hospital, Athens Greece

**Keywords:** Pertrochanteric fractures, Hip nailing, Short nails, Long nails

## Abstract

*Purpose*: To compare short with long intramedullary hip nailing for elderly patients with unstable pertrochanteric fractures.

*Methods*: We prospectively studied 50 patients (33 women, 17 men; mean age, 80 years; range, 74–93 years) with unstable pertrochanteric fractures admitted and treated with a short (group A) or a long (group B) intramedullary hip nail from January 2013 to 2017. The patients were randomly allocated into each group according to their order of admission. The mean follow-up was 2 years (range, 1–5 years). We evaluated operative time, function, fracture healing, varus/valgus loss of reduction, and distance between the distal line of the fracture and the distal locking screw of the nail.

*Results*: Operative time was significantly shorter in group A. Function, fracture healing and varus/valgus loss of reduction was similar between the two groups. The mean distance between the distal fracture line and distal locking screw was 7.2 cm (range, 3–10 cm) in patients of group A; in all patients of group B, an appropriate nail length was chosen so that the distal locking screw was inserted at least 3 times the diameter of the bone at the distal fracture line. Complications included periprosthetic fracture (one patient of group A), and *z*-effect phenomenon (one patient of group B); complications rate was similar between the two groups.

*Conclusion*: Short intramedullary hip nailing is associated with similar function and complications, but shorter operative time compared to long intramedullary hip nails for patients with unstable pertrochanteric fractures.

## Introduction

Pertrochanteric hip fractures in the elderly are very common low energy injuries as a result of osteoporosis [1,2]. With rising life expectancy, it is estimated that the incidence of hip fractures will rise to approximately 6.26 million by 2050 [1]. The need for surgical treatment and early mobilization of the patients with these fractures is well documented; the goal of treatment is not only to reduce the morbidity/mortality rates associated with prolonged immobilization, but also to improve the functional result in terms of malunion and mobility [2]. Several classifications have been described for the pertrochanteric fractures. The classification proposed by AO/OTA into stable and unstable fractures is very useful in the clinical setting. The unstable pertrochanteric fractures (AO/OTA 31A2 and 31A3) and especially the reverse oblique variants (AO/OTA 31A3) have specific biomechanical patterns such as the lack of medial buttress that make their treatment challenging [3,4].

There are conflicting reports regarding the optimal osteosynthesis device for pertrochanteric fractures [5–9]. Various extramedullary (plate and screws constructs) and intramedullary (intramedullary hip nails) osteosynthesis implants have been described, however, the choice of fixation method remains a focus of dispute among orthopaedic trauma surgeons [5,6]. As a general rule, intramedullary nails are more preferable in unstable pertrochanteric femoral fractures due to their biomechanical and technical characteristics [7]. In contrast, extramedullary implants seem to have a biomechanical disadvantage when compared with intramedullary nails because the load transfer in the proximal femur is predominantly shared through the femoral calcar. Additionally, intramedullary nails are more stable under load with a shorter lever arm, therefore, the distance between the hip joint and the nail is reduced compared with that for a plate [8].

There is limited information regarding the optimal length of the intramedullary hip nails for unstable pertrochanteric fractures; although long nails have been proposed for unstable fractures, it is still not proven their superiority over short nails [9–11]. Several studies have reported positive outcomes with short versus long intramedullary hip nails for unstable pertrochanteric fractures; however, conflicting issues remain [10,11]. Therefore, we performed this study on elderly patients with low energy, unstable pertrochanteric hip fractures to compare the outcome (operative time, fracture healing, function and complications) of treatment with short versus long intramedullary nailing.

## Materials and methods

We prospectively studied 50 elderly patients with low energy unstable (AO/OTA 31-A2 and 31-A3) pertrochanteric hip fractures admitted and treated at the authors' institution from 2012 to 2016. There were 33 women and 17 men, with a mean age of 80 years (range, 74–93 years). Patients with unstable pertrochanteric pathological fractures as well as those with a previous pertrochanteric fracture of the contralateral hip were excluded. Patients were randomly allocated into two groups according to their order of admission; group A included 25 patients (mean age, 81 years; range, 74–92 years) that were treated with a short intramedullary hip nail, and group B included 25 patients (mean age, 79 years; range, 74–93 years) that were treated with a long intramedullary hip nail. The mean follow-up was 2 years (range, 1–5 years); 35 patients had a minimum follow-up of 1 year. No patient was lost to follow-up; all patients gave written informed consent for surgical treatment and for their data to be included in this study. This study was approved by the Institutional Review Board/Ethics Committee of the authors' institution.

**Figure 1:**
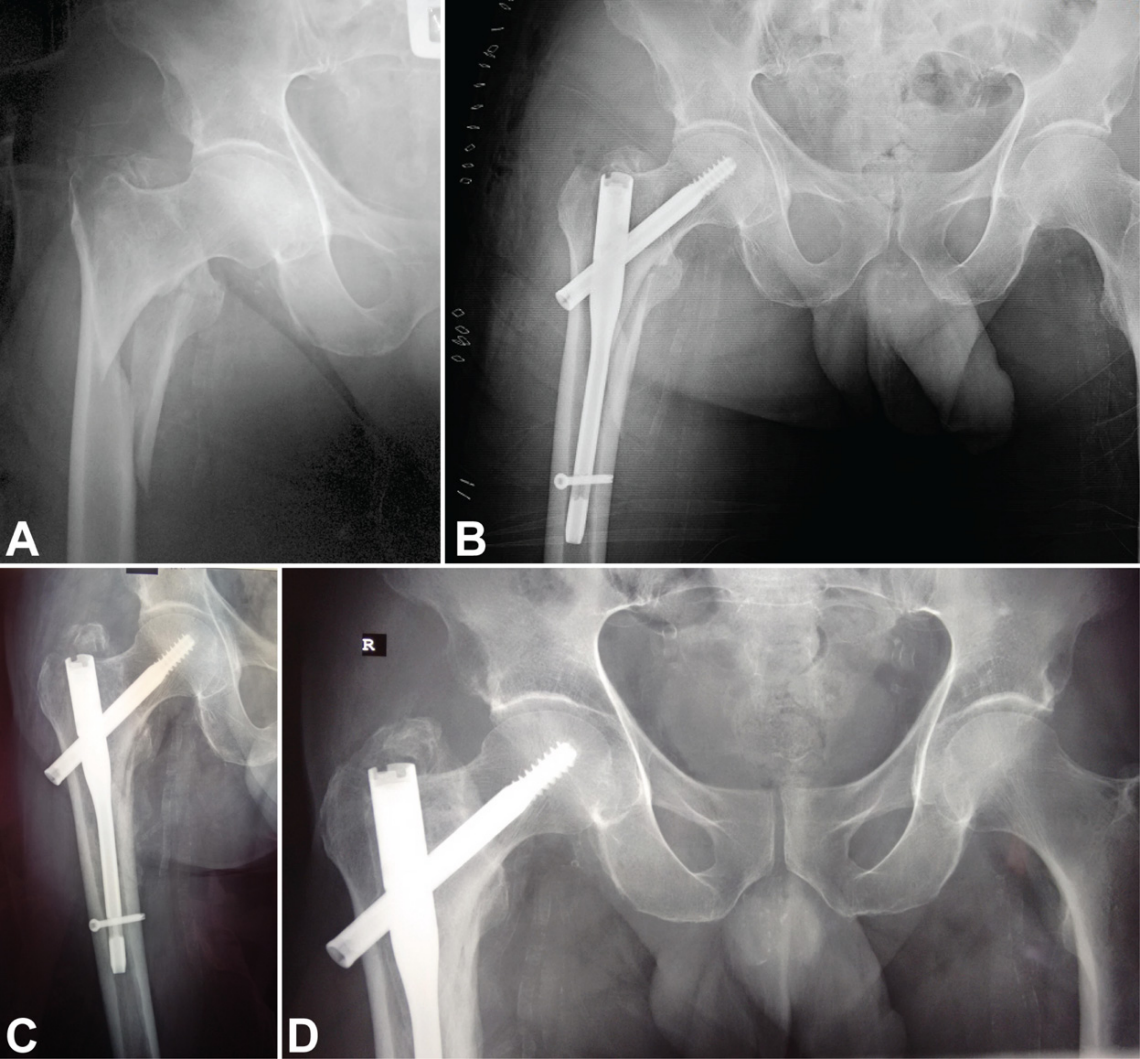
(A) Anteroposterior radiograph of the right hip of a 74-year-old man with an unstable pertrochanteric fracture (AO/OTA 31A2.2). (B) Postoperative radiograph after closed reduction and fracture fixation with a short intramedullary hip nail; the distance between the distal fracture line and the distal locking screw is 3 cm. (C) Anteroposterior radiograph of the right hip at 3-month follow-up shows fracture healing; the patient was weight-bearing with a cane. (D) Anteroposterior radiograph of the right hip at 12-month follow-up shows complete fracture healing.

**Figure 2:**
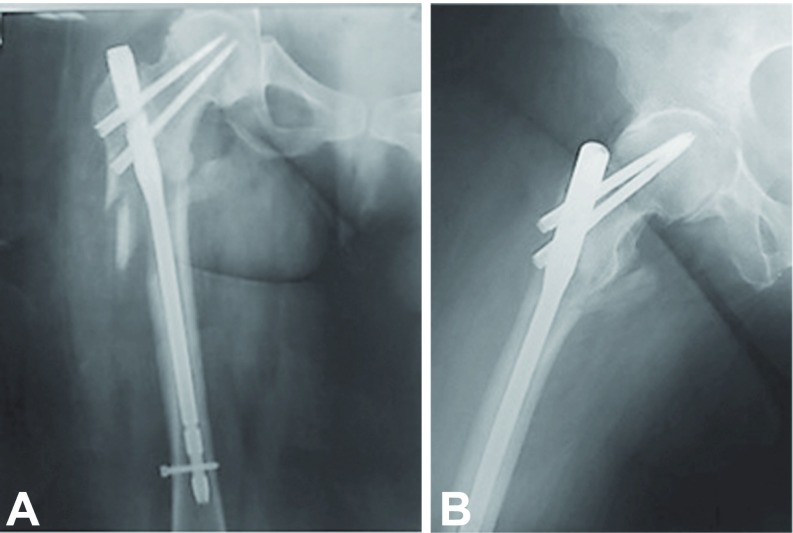
(A) Anteroposterior radiograph of the right hip of an 80-year-old woman with an unstable pertrochanteric fracture (reverse oblique, AO/OTA 31A3.3) of the left hip treated with long intramedullary hip nail. (B) Lateral radiograph of the right hip at 3-month follow-up shows fracture healing; the patient was weight-bearing with a walker.

All patients had preoperative evaluation and management of potential comorbidities including coronary artery disease and chronic heart failure (five patients), endocrinopathies such as diabetes mellitus and hyperthyroidism (13 patients) and chronic renal failure (two patients) by the same team of anesthesiologists in charge for preanesthetic/operative evaluation of orthopaedic patients. In group A patients, the Affixus Hip Fracture Nail System^®^ (Zimmer BIOMET, Warsaw, IN, USA) was used. This system includes a standard nail of 180 mm length and minimum diameter of 9 mm, a single head screw of 10.5 mm width, an optional antirotation screw, and two distal holes for static and dynamic locking (Figure 1). In group B patients, the OrthofixVeroNail Trochanteric Nail^®^ (Orthofix, Verona, Italy) was used. This system includes nails with length from 280 to 440 mm, a minimum diameter of 10 mm, two head screws in a parallel (lag) or convergent (locked) configuration, and two distal holes for static and dynamic locking (Figure 2). In all patients of group B, a convergent head screw configuration was used, and an appropriate nail length was chosen so that the distal locking screw of the nail to extend at least 3 times the diameter of the bone at the distal line of the fracture. In all patients of both groups, static distal locking of the nails was done.

All patients were operated under spinal anesthesia in a supine position on a fracture table. Closed reduction with traction was done and nailing of the fracture was performed according to the standardized technique in all patients. A suction drain was not applied in any of the patients in this series. The operative time was recorded. Postoperatively, all patients were mobilized in the sitting position on the second postoperative day, and allowed protective, partial (approximately 30% of body weight) weight bearing with a walker for 1 month, allowing weight bearing with the walker or canes as tolerated, thereafter.

Routine postoperative clinical and radiographic examination [12,13] was done at 1, 3, 6 and 12 months, and then annually until the last follow-up, for the purpose of this study (study end point). Clinical examination included evaluation of function by the time for weight bearing with the use of a single crutch or cane, leg length discrepancy and Trendelenburg gait. Leg length discrepancy was measured clinically from the anterior superior iliac spine to the tip of the medial malleolus, and compared with the contralateral extremity. Radiographic examination included evaluation of fracture healing, varus/valgus loss of reduction on anteroposterior radiographs, and distance between the distal line of the fracture and the distal locking screw of the nail (group A patients). We defined radiographic fracture healing as the evidence of trabeculation and cortical bridging in at least three cortices [12]. We compared the clinical and radiographic variables between the two groups of patients at follow-up with Student's *t* test for quantitative data and chi-square for qualitative comparative data. Data were recorded in a Microsoft^®^ Excel^®^ 2010 spreadsheet (Microsoft Corporation, Redmond, WA, USA) and analyzed using IBM^®^ SPSS^®^ Statistics version 24 software (IBM Corporation, Armonk, NY, USA).

## Results

Operative time was significantly shorter in group A (*p* *=* 0.001); the mean operative time was 41 min (range, 20–51 min) in group A compared to 54 min (range, 35–70 min) in group B.

Function was similar between the two groups (*p* = 0.910). The mean time for weight bearing with a single crutch or cane was 7.85 weeks (range, 6–9 weeks) for group A compared to 7.31 weeks (range, 6–9 weeks) for group B; eight and three patients of group A experienced a Trendelenburg gait at 3- and 12-month follow-up compared to seven and three patients of group B, respectively; three patients of group A and two patients of group B experienced a leg length discrepancy of <1 cm.

Fracture healing was similar between the two groups. All patients experienced complete fracture healing; the mean time for fracture healing was similar between the two groups (mean, 11 weeks; range, 9–12 weeks; *p* = 0.440).

Varus/valgus loss of reduction was similar between the two groups (*p* = 0.450); none patient of any group experienced varus/valgus loss of reduction within the period of this study.

In patients of group A, the mean distance between the distal fracture line and distal locking screw was 7.2 cm (range, 3–10 cm). In all patients of group B, an appropriate nail length was chosen so that the distal locking screw was inserted at least 3 times the diameter of the bone at the distal fracture line.

Complications were similar between the two groups; one patient of group A experienced a periprosthetic fracture at the tip of the nail that was revised with a longer nail, and one patient of group B experienced a *z*-effect phenomenon 3 months after treatment that was revised with hip hemiarthroplasty. By direct comparison and analysis of the complications related to the length of the nails (periprosthetic fracture in one patient of group A) and excluding complications related to the type of the nails used (*z*-effect phenomenon in one patient of group B), the complications rate was significantly higher in group A patients (*p* = 0.061).

## Discussion

Although there are no obvious advantages of the intramedullary hip nails compared to plate and sliding screw constructs for stable pertrochanteric fractures, the use of intramedullary hip nails for unstable pertrochanteric fractures seems to have many advantages including biomechanical superiority, construct stability, higher fracture healing rates, better function for the patients, and cost-effectiveness [14–18]. Therefore, intramedullary hip nailing has become the treatment option of choice for pertrochanteric fractures in general and unstable variants specifically [17]. However, there is limited information regarding the use of short or long hip nails for these fractures types. Therefore, we performed this prospective study to evaluate the outcome (function, fracture healing and complications) of the patients with unstable pertrochanteric fractures treated with short versus long intramedullary nailing. Our results showed similar function of the patients, shorter operative time with short intramedullary hip nailing, and similar fracture healing and complications. Therefore, based on these findings, although on a small cohort of patients, short hip nailing should be considered an appropriate treatment option for patients with unstable pertrochanteric fractures. In line with the related literature [19], the shorter operative time with short intramedullary hip nailing should be considered a benefit for the patients; radiation exposure for the surgeons and the patients is also reduced [19].

We see three limitations in this series. First, the number of patients included in this series is relatively small. We acknowledge this limitation; however, although there is no proven evidence so far in the literature against short nails for treating unstable pertrochanteric fractures, this prospective comparative study could be useful in this subject. We aim to enlarge this study in the future and reevaluate the findings again. Second, we did not use a robust method of randomization for a formal randomized controlled study, and did not perform a power analysis at baseline for the appropriate number of study patients. In this regard, our results should be considered with caution. Instead, we aimed to include all our patients with an unstable pertrochanteric fracture admitted and treated at the study period at our institution, and followed these patients over time (prospectively). Third, we used different types of nails between the two groups of patients. At the time period of this study, only dual head screw long hip nails were available at our hospital, therefore, this type of long hip nails was used for comparison in this study. We consider this an important limitation; however, we do not believe that the head screws significantly alter the function of the patients and the healing of the pertrochanteric fractures [18]. In contrast, we do believe that the periprosthetic fracture that occurred in one patient of group A should be related to the nail length and the *z*-effect phenomenon that occurred in one patient of group B should be related to the type of the nail. Definitely, if we had used similar types of nails in both groups our analysis and results would have been more useful.

Recent studies did not report a difference in function between patients with pertrochanteric fractures treated with either a short or a long intramedullary hip nail [4,20]. Similarly, other studies [5,11] reported similar function, fracture healing and revision rates with a short or a long intramedullary hip nail, and a shorter operative time with a short hip nail. Additionally, these studies emphasize on a higher rate of complications with short versus long intramedullary hip nailing [4,5,11,20]. An overall rate of periprosthetic femoral fractures ranging from 0 to 20% has been reported in patients treated with short intramedullary hip nails for pertrochanteric fractures [4]; some authors believe that long intramedullary nails protect the entire femur from periprosthetic fractures [4] while others report no difference in complications [20]. We concur with these studies reporting periprosthetic fractures as a complication of short intramedullary hip nailing [4,5,11,20]; periprosthetic fractures after short intramedullary nailing have been reported for all types of hip fractures, and the accurate fracture pattern seems to be an independent factor. Although our results showed similar complications between the two groups of patients, the periprosthetic fracture should probably be considered a complication related to the length of the nail. However, we have acknowledged the small number of patients in our study. Therefore, we suggest short intramedullary nail for unstable pertrochanteric fractures and we recommend long nails for protection of the total femur to avoid periprosthetic fractures.

Varus/valgus loss of reduction is an important factor that may affect the outcome and increase the complications rates for the patients with either stable or unstable pertrochanteric fractures [13,21–23]. Trendelenburg gait, although often a result of abductor muscles injury during the approach and nail insertion, it may also be related to varus malreduction of the fracture from failure to reduce the fracture with traction or after nail insertion through a very lateral entry point at the great trochanter. Varus malreduction produces elevation of the tip of the great trochanter relative to the center of the femoral head and shortening of the abductors lever arm, resulting in abductor insufficiency. Although surgical tips and techniques have been reported to avoid mistakes in reduction and surgical treatment [13,22,24], the surgeons should not accept malreduction, especially in varus, as this can also increase osteosynthesis failure rates [21]. In the present study, there was no difference in loss of reduction between the two groups of patients. Probably, this should be attributed to the optimal fracture reduction and surgical technique.

## Conclusion

Short intramedullary hip nailing is associated with similar function and complications, but shorter operative time compared to long intramedullary hip nailing for patients with unstable pertrochanteric fractures.

## Conflict of interest

The authors declare that they have no conflicts of interest in relation to this article.
